# Efficacy of Injury Prevention Using Functional Movement Screen Training in High-School Baseball Players: Secondary Outcomes of a Randomized Controlled Trial

**DOI:** 10.3390/healthcare10122409

**Published:** 2022-11-30

**Authors:** Kenta Suzuki, Yasuaki Mizoguchi, Fumihiko Kimura, Yutaka Sawada, Kiyokazu Akasaka

**Affiliations:** 1 Graduate School of Medicine, Saitama Medical University, 981 Kawakado, Moroyama 350-0496, Saitama, Japan; 2 Kimura Orthopedic Clinic, 689-4 Harajima, Kumagaya 360-0811, Saitama, Japan

**Keywords:** functional movement screen, high-school baseball player, injury prevention, youth sports

## Abstract

This study of a randomized controlled trial aimed to clarify the effect of functional movement screen (FMS) training on the FMS score and the number of injuries in high-school baseball players. Accordingly, 71 high-school baseball players (age: 15–17 years) were randomized into an intervention group (*n* = 37; FMS training 4 times per week for 12 weeks on the ground of each team) or a control group (*n* = 34; team practice without limits). No significant differences were observed in terms of the participant characteristics of the two groups. The FMS score of the intervention group significantly increased after 12 weeks of training compared with the control group. However, there was no difference between the groups in terms of the FMS score after 24 weeks. Injuries in the intervention group were significantly reduced after 24 weeks. The time lost due to noncontact injuries (12 weeks/follow-up) was 56.5 h/113 h in the intervention group and 33 h/325.5 h in the control group. Injuries were found all over the body. Based on these results, FMS training was proven to reduce injury in high-school baseball players. Although continued training is required to improve FMS scores, the number of injuries decreased after training.

## 1. Introduction

Throwing in baseball is a complex and demanding movement that produces high segmental velocities and joint forces [1]. Therefore, many reports focus on upper extremity injuries [2,3,4]. It is a persistent problem in playing baseball. However, baseball requires not only repetitive motions, such as pitching and batting, but also various motions, such as base-running and defensive motions [5]. In pitching and batting, the coordination of the lower extremity, trunk, and upper extremity is important because dysfunctions of the trunk and lower extremity and inefficiency of movement lead to disabilities [6,7]. Baseball injuries continue to be a significant problem for athletes, coaches, parents, and sports medicine providers. Given the importance of examining not only the upper extremity but also the trunk and lower extremity for disability prevention, a comprehensive evaluation of the body is necessary.

Functional movement screens (FMS) have been incorporated to assess whole-body movement patterns and left–right asymmetry. FMS is a screening tool that evaluates the whole body [8,9] and is used as a screening process considered necessary for sports injury prevention and recovery [10,11,12]. The FMS consists of seven functional tasks involving the trunk and upper and lower extremity movement patterns. Each task is scored according to ability, with a maximum of 3 points indicating healthy task completion (a total of 21 points indicates a perfect aggregate score for all tests) [8,9]. In general, it has been reported that an FMS score of 14 points or less increases the risk of injuries [11,13]. On the contrary, the FMS score may not be useful in predicting injury risk [14,15], and there is no consensus.

In Major League Baseball (MLB) and Minor League Baseball (MiLB), the prevalence of ulnar collateral ligament (UCL) reconstruction in players is 13% [16], with 25% of MLB and 15% of MiLB pitchers having UCL surgery; UCL injuries are likely to end a player’s season [17]. Among high school baseball players, 39.6% of shoulder injuries and 56.9% of elbow injuries are caused by overuse in pitchers [2], and 49.7% of players experience low back pain, not only in the upper extremities [18]. Therefore, the focus of prevention and treatment should be expanded to adolescents, and it is important for long-term baseball to acquire functional movements in baseball from adolescence and reduce the stress on the whole body. It is also very useful for team trainers and coaches to investigate the number of injuries by assessing functional movement and training with FMS.

A study reported that FMS scores are improved by FMS training for high-school baseball players [19]. It is important to demonstrate in the field that FMS training not only improves FMS scores but also reduces the number of injuries and time lost due to injury. However, few studies have reported the injury prevention effect of FMS training, and no study has examined how much it affects baseball practice time.

Therefore, this study aimed to clarify whether FMS training for high-school baseball players would reduce the number of injuries and lost time. If the number of injuries and loss time of athletes who underwent FMS training decreases, it can be judged that it is worth adopting as injury prevention training. The hypothesis of this study is that a high-school baseball player on FMS training will reduce the number of injuries and lost time.

## 2. Materials and Methods

### 2.1. Study Design

A detailed protocol of the RCT has been published previously [19]. This parallel randomized controlled clinical trial was registered at the University Hospital Medical Information Network Center, Tokyo, Japan (UMIN000027553).

### 2.2. Participants

The study participants were recruited from April 2017 to June 2017. The participants of this randomized controlled clinical trial in high-school male baseball players were selected from the top 32 schools of the Saitama Prefecture High School Baseball Championship and agreed to participate in this study. This is to equalize the competition level and practice time. The inclusion criteria were high-school male baseball players who were not injured at the beginning of the study. Players who had difficulty practicing or intervening because of injury were excluded. All athletes were randomly grouped into the intervention and control groups, as each high school practice may influence the results. For randomization, the envelope method was employed, i.e., opaque envelopes contained the assignments to the intervention or control groups. One physiotherapist (K.S.) selected the participants, generated the randomization sequence, and assigned the players to each group.

### 2.3. Sample Size

We calculated the sample size required to achieve a statistical significance of 80% power (1−β) before study initiation. An application for power analysis (G*Power 3.1.9.4 provided by Heinrich-Heine-Universität Düsseldorf, http://www.gpower.hhu.de/ accessed on 1 March 2017) was used to calculate the sample size. The Chi-square test was performed with an effect size of 0.3 (a = 0.05, 1 − β = 0.8) to compare the number of disorders in both groups, resulting in 88 cases. Therefore, we tried to recruit at least 88 subjects prior to the beginning of this study.

### 2.4. Questionnaire

Participants recorded their age, height, weight, years of baseball experience, dominant hand, and field position in the questionnaire. The dominant hand was determined as the arm used for pitching.

### 2.5. Outcomes

This study is an analysis of the secondary outcomes of the developed RCT. The primary outcome variable was the FMS score, which has been previously published [19].

#### 2.5.1. Players Record

From September 2017 to March 2018, we calculated the number of injuries (contact and noncontact), their types, and time lost in practice due to injury as the primary outcomes. We counted an injury as a problem, such as pain that occurred during a baseball practice or game [20]. Since the high school that was targeted in this study did not have an athletic trainer, the evaluation and intervention by a physiotherapist were counted as one occurrence of injury in this study. During the study, the players recorded the time lost due to injury and the type of injury. An elected representative from each high school reported to the physiotherapist when team injuries occurred and ensured that these were properly documented.

#### 2.5.2. FMS

**Figure 1:**
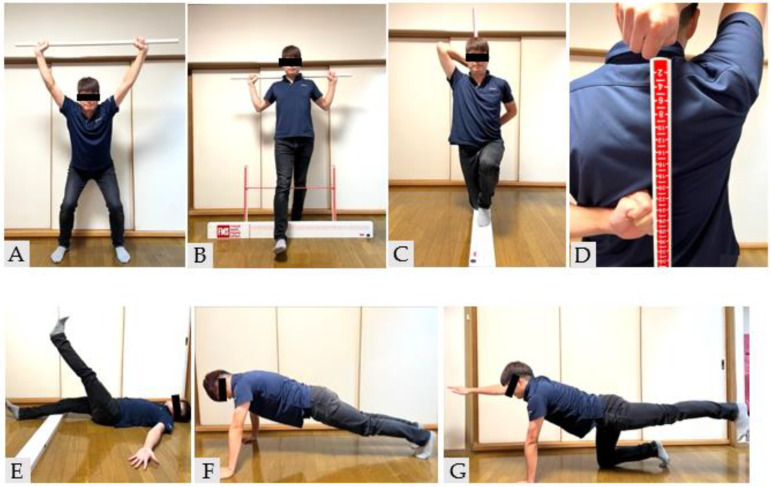
Functional movement screen. (**A**) Deep squat, (**B**) hurdle step, (**C**) inline lunge, (**D**) shoulder mobility, (**E**) active straight leg raise, (**F**) trunk stability push-up, and (**G**) rotary stability.

The FMS score was measured as the secondary outcome and in the same manner as previously stated [19]. This screening test is based on the method proposed by cook et al. [8,9]. The FMS comprised seven tasks, including deep squat (Figure 1A), hurdle step (Figure 1B), inline lunge (Figure 1C), shoulder mobility (Figure 1D), active straight leg raise (Figure 1E), trunk stability push-up (Figure 1F), and rotary stability (Figure 1G) [8,9].

Measurements were taken before the intervention, 12 weeks after the intervention, and after 12 weeks of follow-up. One FMS-trained physical therapist measured all items to reduce measurement bias and was blinded.

### 2.6. Intervention

All players in the control group performed their normal team practice; they did not perform FMS training, and there were no limits to the practice. All players in the intervention group performed FMS training 4 times a week for 12 weeks for tasks with an FMS score of ≤2 points. Thereafter, no FMS training was performed for another 12 weeks, and normal exercises were performed the same as in the control group. A detailed protocol (randomization and intervention method) for FMS training has been previously published [19].

### 2.7. Statistical Analysis

Data were analyzed with IBM SPSS Statistics for Windows version 25.0 (IBM Japan, Tokyo, Japan). Unpaired t-tests were used to compare the characteristics of the intervention and control groups. The Mann–Whitney U test was used to compare the FMS scores at each time period between the two groups. Dunnett’s test was performed for within-group comparisons of the FMS score at pre-intervention, after 12 weeks, and after 24 weeks. The number of injuries was classified into contact and noncontact injuries, and the Chi-square test was performed for each group after 12 and 24 weeks. Ancillary analyses were performed using the Mantel–Haenszel test to identify items that could influence the relative risk (RR). RR was calculated based on the number of injuries. Lost time was calculated as 0–12 and 12–24 weeks, respectively, when baseball was not played because of injury. Statistical significance was set at *p* < 0.05.

### 2.8. Ethical Considerations

To participate in this study, written information about the study was provided, and consent was obtained from all players, their parents, principals of their high schools, and the head of the baseball club. After confirming that the players understood the study, we sought consent from their parents. This study was approved by the ethics review committee of the Faculty of Health and Medical Care of Saitama Medical University (M-75).

## 3. Results

From April 2017 to June 2017, a total of 71 baseball players aged 15–17 years were recruited from four high schools in the Saitama Prefecture. They were then randomly assigned to an intervention group (*n* = 37) and a control group (*n* = 34). No significant differences were found in participant characteristics between the two groups (Table 1). The intervention took place from September 2017 to November 2017, i.e., for 12 weeks. In this study, most endpoints were obtained, and no serious adverse effects occurred. The compliance rate based on the questionnaire survey and FMS training in the intervention group was 100%. Owing to the lower-than-planned number of participants, a post hoc power analysis was performed.

### 3.1. Primary Outcome

#### 3.1.1. Number of Injuries

After 12 weeks, a significant difference was found between the two groups in the total number of contact and noncontact injuries (*p* = 0.83, 1 − β = 0.08) and only noncontact (*p* = 0.71, 1 − β = 0.11). After 24 weeks, the sum of contact and noncontact (intervention, 3; control, 11) (*p* = 0.01, 1 − β = 0.99) and noncontact only (intervention, 3; control, 10) (*p* = 0.02, 1 − β = 0.99) were significantly decreased in the intervention group (Table 2).

#### 3.1.2. RR and Lost Time to Injuries

The times lost to contact and noncontact injuries by 12 weeks were 72.5 h and 111 h in the intervention group and control group, respectively, with a RR of 0.98. The times lost due to noncontact injuries alone were 56.5 h and 33 h, with RR of 0.98, respectively. The times lost to contact and noncontact injuries from 12 to 24 weeks were 113 h and 332.5 h in the intervention group and control groups, respectively, with a RR of 1.36. The times lost to noncontact injuries alone were 113 h and 325.5 h, with RR of 1.30, respectively (Table 2). Contact injuries were fractures, and noncontact injuries were pain in the shoulder joints, lumbar vertebrae, and ankle joints (Table 3). Injuries counted in lumber, shoulder, and ankle were areas where players complained of pain during baseball movements. Fractures in the intervention group at 0–12 weeks were humerus fractures due to player-to-player contact and finger fractures due to the same cause in the control group. 

#### 3.1.3. Ancillary Analysis

Breslow–Day and Mantel–Haenszel tests were performed to eliminate the confounding effects of position on failure incidence. The Breslow–Day test χ^2^ value was 2.63 (*p* = 0.621) for all disorders from 0 to 12 weeks, indicating similar levels of RR for each stratum. The estimated common odds ratio for the Mantel–Haenszel test was 1.31 (95% CI = 0.24–7.10). Also tested in the same way, the values were 3.27 (*p* = 0.36) and 0.9 (95% CI = 0.13–6.74) for noncontact injuries by 0–12 weeks, 4.86 (*p* = 0.56), 5.16 (95% CI = 1.09–24.51) for all injuries by 12–24 weeks, and 4.44 (*p* = 0.62) and 4.85 (95% CI = 0.99–23.73) for noncontact injuries by 12–24 weeks. In addition, Breslow–Day and Mantel–Haenszel tests were also performed for years of experience, showing the overlap of RRs within each factor. This analysis yielded χ^2^ of 3.65 (*p* = 0.30) and 0.77 (95% CI = 0.18–3.39) for all injuries by 0–12 weeks, 4.32 (*p* = 0.12) and 0.88 (95% CI = 0.12–6.55) for noncontact injuries by 0–12 weeks, 6.47 (*p* = 0.17) and 4.67 (95% CI = 1.03–21.24) for all injuries by 12–24 weeks, and 5.77 (*p* = 0.22) and 4.23 (95% CI = 0.90–19.88) for noncontact injuries by 12–24 weeks. Collectively, our analysis showed that the confounding factors did not influence the main outcome of the study.

### 3.2. Secondary Outcome

#### FMS Score

Intragroup and intergroup comparisons of the FMS scores are shown in Table 4. Baselines were equal, with no significant difference in preintervention FMS scores. In the group comparison after 12 weeks, the intervention group (17.5 ± 1.5) had significantly increased total FMS score compared with the control group (14.7 ± 2.3); however, after 24 weeks, no significant difference was found between the intervention group (15.3 ± 2.3) and the control group (15.5 ± 1.6). The FMS scores of the intervention (pre, 13.7 ± 2.6) and control (pre, 13.8 ± 2.7) groups increased after 12 and 24 weeks. The subitems of the FMS are shown in Table 4. 

## 4. Discussion

In this study, FMS training was found to reduce the number of injuries and improve FMS scores. No difference was found in the incidence of injuries between the two groups after 12 weeks; however, it was significantly lower in the intervention group after 24 weeks in the total of contact and noncontact or noncontact injuries alone. The RR was 1.36 for all injuries and 1.3 for noncontact injuries, indicating a 1.3-fold higher chance of injury compared to the control group. Regarding disability-prevention programs, the effects of measures such as stretching, physical function, and movement exercises have been presented [21,22]. In this study, the number of injuries did not decrease after 12 weeks of intervention; however, good movement patterns and kinetic chain were acquired after 12 weeks of FMS training. Therefore, continuous training was considered to improve movements after 12 weeks and decrease the number of injuries after 24 weeks. In addition, since the number of injuries, including contact, was reduced in the intervention group, the improvement of the movement pattern possibly contributed to the process until the contact injury. On the contrary, no immediate effect was found on the reduction of the number of injuries. Duke et al. reported that athletes with low FMS scores may be at higher risk of contact injuries due to a history of injuries that may underlie their low FMS scores [23]. Measurement of FMS may predict contact injuries, but it may not predict and prevent all contact injuries because player-to-player contact is not the only cause. The follow-up in this study was only up to 24 weeks; thus, it is unclear how long the effect of reducing the incidence of injuries will last. If it contributes to improving FMS scores and reducing the number of injuries, FMS training may be incorporated from an early stage and aim for future injury prevention. In addition, the time lost to noncontact injury was more at 12 weeks in the intervention group; however, it increased in the control group at 24 weeks. Not only in baseball, a sports injury is a very important issue because it affects not only the lost time but also the economic cost loss [24]. This loss of time was also attributed to the effect of 12 weeks of training, as was the number of injuries. However, a direct comparison cannot be made regarding the lost time, as the injury site differs from player to player and is related to the severity of the injury. It appears necessary to comprehensively evaluate the whole body when considering injury prevention in baseball.

Consistent with our results, Bodden et al. reported that FMS training improved the total FMS scores at 4 and 8 weeks in mixed martial arts athletes [25], and Kiesel et al. reported that FMS training improved FMS scores and asymmetry in football players [12]. FMS training is important for improving the functional patterns of movement, movement asymmetry, and energy transfer effect of the kinetic chain [12,26]. This study also appears to have had similar effects on each item, suggesting that continuous interventions rather than short-term interventions are necessary to maintain the FMS score. Studies have reported that training using cable machines and kettlebells improves FMS scores, but this study showed that simple FMS training using towels and cushions is effective. However, both the intervention and control groups had improved FMS scores compared with before the intervention. In the comparison between groups at 12 weeks, the intervention group significantly improved. These were reflected in the FMS score by functional exercises, such as strength training, stretching, and baseball-specific technical practice in regular baseball practice. This indicates a further improvement effect caused by the intervention.

It is possible that structured intervention improved functional movement as measured by the FMS and that off-season programs were able to normalize the movement pattern and asymmetries [12]. Incorporating recommended training into a standard strength and conditioning program partially improved functional movement compared to a strength and conditioning program alone [27]. Therefore, this study also suggests that training for the FMS subitems partially improved functional movements.

In this study, FMS training was performed on high school baseball players for 12 weeks. However, in previous papers, a standardized off-season intervention program was performed to improve FMS in professional football players for 7 weeks [12], martial arts athletes for 4 weeks [25], female netball players for 6 weeks [27], preprofessional young football players for 12 weeks [28]. These imply differences in gender, sports, intervention duration, and intervention frequency; it may not be concluded that FMS training alone contributed to FMS scores.

On the other hand, FMS training for high school baseball players improved muscle strength and flexibility and enhanced core stability, contributing to improved body balance and overall strength [29]. Considering that the participants’ characteristics were closest to this study, improvements in muscle strength and flexibility may have influenced improvements in FMS scores in this study as well, but we cannot be sure because we did not measure them.

This study has certain limitations. The study participants were limited to adolescent baseball players, making it difficult to generalize this result for baseball players of all ages. However, considering that lower extremity and trunk injuries are more common in adults [3,17,30], we believe that it is reasonable to implement preventive measures during adolescent athletes. Furthermore, FMS training was given to low-scoring FMS items, and no attempt was made to identify specific, detailed causes. Future studies should investigate the causes of movement dysfunction individually and present a more specific approach. Regarding the sample size, although the planned number of participants was not reached, a post hoc power test was performed to confirm that the FMS total score and number of injuries 1−β were ≥0.8. In addition, the off-season when the intervention was performed is the time of the year when there is less baseball technical practice; thus, it may be necessary to reconsider the timing for baseball movement injuries. As mentioned above, the severity of the injuries was not taken into account; in the future, we would like to investigate the relationship by severity and consider the prediction of injury by FMS and the adaptation of training content. 

## 5. Conclusions

The results of this study showed that 12 weeks of FMS training reduced the number of injuries after 24 weeks in high-school baseball players, regardless of their position or years of experience. The control group was also 1.3 times more likely to be injured than the intervention group, and the injury occurred not only in the upper extremities but also in the trunk and lower extremities. Therefore, a comprehensive systemic assessment is necessary, and FMS training may be important for injury prevention. However, there is no fixed consensus on the competitions in which FMS training should be employed and the duration of the intervention, and more research is needed.

## Figures and Tables

**Table 1:** Subject characteristics. (*n* = 71).

		Intervention (*n* = 37)	Control (*n* = 34)	*p*-Value	1−β
Age (years)		16.0 ± 0.1 (15–17)	15.8 ± 0.1 (15–17)	0.17	1.00
Height (cm)		169.3 ± 0.9 (156–181)	169.7 ± 1.0 (158.6–179)	0.76	0.399
Body weight (kg)		63.5 ± 1.4 (43–84)	62.1 ± 1.2 (49–80)	0.41	0.99
Body mass index		22.1 (17.7–28.7)	21.5 (18.1–26.1)	0.27	0.18
Experience as a baseball player (years)		7.7 ± 0.3 (2–10)	7.3 ± 0.3 (3–10)	0.37	0.99
Dominant hand (*n*)	Right/left	34/3	28/6	0.29	0.66
Position (*n*)	Pitcher	6	9	0.38	0.38
	Catcher	3	4	0.70	0.09
	Infielder	16	14	1.00	0.06
	Outfielder	12	7	0.29	0.34
mean ± SD (min–max)					

**Table 2:** Number of injuries, time lost to sports injury, and relative risk.

	None	Number of Injuries (*n*)	Lost Time (h)	Injury Rate RR (95% CI)	*p*-Value	1 − β
[0–12 weeks]						
All injuries						
Intervention	32	5	72.5	0.98 (0.82–1.17)	0.83	0.08
Control	30	4	111			
Noncontact						
Intervention	34	3	56.5	0.98 (0.86–1.11)	0.71	0.11
Control	32	2	33			
[12–24 weeks]						
All injuries						
Intervention	34	3	113	1.36 (1.06–1.75)	0.01	0.99
Control	23	11	332.5			
Noncontact						
Intervention	34	3	113	1.30 (1.03–1.65)	0.02	0.99
Control	24	10	325.5			

RR, relative risk.

**Table 3:** Injury sites up to 12 weeks and from 12 to 24 weeks.

	Intervention (*n*)		Control (*n*)	
	Contact	Noncontact	Contact	Noncontact
[0–12 weeks]				
Fracture	1		1	
Lumber		1		1
Shoulder		1	1	1
Ankle	1	1		
[12–24 weeks]				
Lumber				5
Shoulder		3	1	5

**Table 4:** Comparison of the FMS score between the intervention and control groups.

	Group	Before the Intervention	After 12 Weeks	After 24 Weeks
Total FMS score	Intervention	13.7 ± 2.6 (8–18)	17.5 ± 1.5 (13–20) ^a,b,c^	15.3 ± 2.3 (9–19) ^b^
	Control	13.8 ± 2.7 (8–18)	14.7 ± 2.3 (9–18) ^b^	15.5 ± 1.6 (12–18) ^b^
Deep squat	Intervention	2 (0–3)	2 (2–3) ^b^	2 (1–3)
	Control	2 (0–3)	2 (0–3)	2 (0–3)
Hurdle step	Intervention	3 (1–3)	3 (1–3) ^a,b,c^	2 (1–3)
	Control	2 (1–3)	2 (1–3)	2 (1–3)
Inline lunge	Intervention	3 (1–3)	3 (2–3) ^a,b,c^	3 (1–3)
	Control	3 (0–3)	2.5 (1–3)	2 (1–3)
Shoulder mobility	Intervention	3 (1–3)	3 (1–3)	3 (1–3)
	Control	3 (1–3)	3 (1–3)	3 (1–3)
Active straight leg raise	Intervention	2 (1–3)	2 (2–3) ^a,b,c^	2 (1–3) ^b^
	Control	2 (1–3)	2 (0–3) ^b^	2 (1–3) ^b^
Trunk stability push up	Intervention	2 (0–3)	3 (2–3) ^a,b,c^	2 (0–3)
	Control	2 (0–3)	2 (1–3)	2 (1–3)
Rotary stability	Intervention	2 (0–3)	2 (2–2) ^b^	2 (1–3)
	Control	2 (1–2)	2 (1–2) ^b^	2 (1–2)

Total FMS score, mean ± SD (min–max); subitems of FMS, median (min–max); ^a^ Significant difference when compared with the control group (*p* < 0.05); ^b^ Significant increase after intervention compared with the values before intervention (*p* < 0.05); ^c^ 1−β value of > 0.8.

## Data Availability

The data presented in this study are available on request from the corresponding author.
